# Factors Associated with Fracture and Migration of Tracheostomy Tube into Trachea in Children: A Case Series

**DOI:** 10.22038/ijorl.2020.44797.2473

**Published:** 2020-11

**Authors:** Pradipta-Kumar Parida, Raja Kalaiarasi, Arun Alexander, Sunil-Kumar Saxena

**Affiliations:** 1 *Department of ENT and Head Neck Surgery, AIIMS Bhubaneswar, Odisha-751019, India.*; 2 *Department of ENT,JIPMER,Pondicherry- 06.*

**Keywords:** Airway, Fracture, Foreign body, Migration, Tracheostomy

## Abstract

**Introduction:**

Tracheostomy is done to bypass the obstructed upper airway. Rare complication of this procedure is the fracture of the tube. Early identification and management of this condition is a great challenge to an otolaryngologist. To study the factors associated with the fracture and migration of tracheostomy tube into tracheobronchial tree in paediatric age group.

**Materials and Methods:**

This study is a case series study conducted on children with a diagnosis of fractured tracheostomy tube presenting as a foreign body airway over five years duration. Data regarding the possible patient and tube factors responsible for the condition were collected and analysed.

**Results:**

Total 11 patients (males-5 and females-6, average age-10.18 years, range 1-15 years) wearing tracheostomy tube for an average period of 2 years (range 3 months-8 years) were included in the study. Aspirated tubes were Jackson’s metallic inner tube, Romson polyvinyl chloride plastic tube and Fuller’s outer tube flange in 5 (45.5%), 4 (36.4%) and 2 (18.1%) patients respectively. The most common fracture site was at the junction between tube and neck plate (90.9%). The most common causes for fracture tube were prolonged use in 10 cases (90.9%), stomal narrowing in 9 cases (81.8%), and infection with peri-stomal granulation tissue in 9 cases (81.8%).

**Conclusion:**

A fractured tracheostomy tube is a rare but preventable late complication of tracheostomy. Appropriate training about proper tracheostomy care, timely check-up of tracheostomy tube for signs of wear and tear, scheduled replacement, regular follow up and awareness may prevention this complication.

## Introduction

Tracheostomy is a commonly performed lifesaving procedure done for bypassing an obstructed upper airway (1-3). Tracheostomy tube (TT) acts as an airway adjunct. TT that are commonly used are metallic tubes and plastic tubes (polyvinyl chloride). The metallic tubes are made of silver, zinc and copper (1-3). Fracture and migration of TT in children may present as tracheobronchial foreign body (FB). This is a rare complication of tracheostomy (2-4) Early identification and management of this condition is a great challenge to an otolaryngologist. Various risk factors such as prolonged use of the same TT, ageing of TT, repeated cleaning and sterilization, alkaline bronchial secretions, tissue reaction to the tube and manufacturing defects have been reported as the causes for the fracture of TT (3-8). Individual case reports of fractured tracheostomy tube (FTT) has been reported in the English literature (2-4). The objective of this study is to examine the patient and tube factors related to fracture and migration of TT.

## Materials and Methods

This case series study was conducted in a tertiary institute in South India on 11 children who presented with TT as a tracheobronchial FB and were removed using rigid bronchoscopy under general anaesthesia over a five-year period. Patient’s demographic details such as age, sex, primary diagnosis, type of TT, duration of using the TT were noted. Once removed, a thorough examination of the migrated part of TT and outer part of TT were done to record the evidence of wear and tear (corrosion). History, findings of tracheostomy stoma and examination findings of FTT were analysed to find out the possible causes for fracture of the tube in each case. TT factors like make of the tube and manufacturing defects were analysed.

Patient factors like duration of use of the same tube (FTT), number of tubes used by the patients, frequency of replacement of the tubes, method of sterilization, use of suction machine at home, history of cough and stomal discharge, frequency and regularity of follow up, awareness about the fracture of TT and need for periodic check-up of TT for signs of wear and tear were also analysed. On clinical examination of the stoma, findings like presence of any stomal infection, peristomal granulation tissue, size of the stroma and presence of peristomal fibrosis were noted. This was a case series of 11 patients not much of statistical methods were used and data were expressed as percentages. Institutional review board approval was obtained for the research work. 

## Results

A total of 11 patients consisting of five male and six female children with the diagnosis of fractured and migrated tracheostomy tube into the tracheobronchial tree were included in the study. The age group of children ranged from 3 months to 15 years. The most common reason for tracheostomy was bilateral abductor palsy (6/11 i.e:54.5%). Other causes were subglottic stenosis due to prolonged intubation and subglottic hemangioma (Table.1).

The make of the aspirated FTTs were Jackson’s metallic inner tube, Romson polyvinyl chloride plastic tube and Fuller’s outer tube flange in five (45.5%), four (36.4%) and two (18.1%) children respectively (Figures:1&2).

The lodgement sites of the fractured tracheostomy tube were found to be different; majority (54.4%) being in the trachea and right main bronchus (Table.1). 

The average duration of TT use was 36 months. The average time duration from the event of aspiration to treatment initiation was 12 hours with a range of under one hour to 32 hours. Usually, parents present for emergency consultations without any delay in case of a missing tracheostomy tube.

The site of fracture for 10 patients were at the junction between the tube and the neck plate. One patient had fracture of one flange of the Fuller’s TT (Table.1). Ten patients (90.9%) had history of prolonged use of FTT. Those patients wearing metallic tubes had only a single tube and the inner tube was cleaned with soap and water and sterilised by boiling. Three out of four patients using Romson tube had 2 tubes and TT was changed once or twice a week (Table 2). None of the TT had been replaced from the time of their first insertion. 

All patients (100%) had history of cough and stomal discharge. Nine patients (81.8%) had stomal narrowing and infection with peristomal granulation tissue (Table.2).

**Table 1:** Detail of paediatric patients with fractured tracheostomy tube

**S. No**	**Age** **(year) /** **Sex**	**Clinical Diagnosis**	**Type of TT and Material make up**	**Duration of wearing the same TT (Months)**	**Fracture site**	**Site of Lodgement of TT**
1.	1/ M	Retropharyngeal abscess with bilateral abductor VC paralysis	Romson’s tube (PVC)	3	Junction between inner tube and neck plate	T just below the stoma
2	6/F	Subglottic stenosis secondary to prolonged intubation	Jackson’s tube (copper, zinc, nickel)	29	Junction between inner tube and neck plate	T and LMB
3	7/F	Subglottic stenosis secondary to prolonged intubation	Romson’s tube (PVC)	10	Junction between inner tube and neck plate	RMB
4	8/M	Congenital subglottic haemangioma	Jackson’s tube (copper, zinc, nickel)	26	Junction between inner tube and neck plate	RMB
5	9/M	Bilateral abductor palsy	Jackson’s tube (copper, zinc, nickel)	24	Junction between inner tube and neck plate	RMB
6	11/F	Bilateral abductor paralysis	Romson’s tube (PVC)	20	Junction between inner tube and neck plate	T below the stoma
7	13/F	Bilateral abductor paralysis	Jackson’s tube (copper, zinc, nickel)	33	Junction between inner tube and neck plate	RMB
8	15/F	Post OP poisoning with subglottic stenosis	Fuller’s tube (copper and Zinc)	48	Junction between inner tube and neck plate	RMB
9	6/M	Subglottic stenosis sencondary to prolonged intubation for retropharngeal abscess	Romson’s tube (PVC)	63	Junction between inner tube and neck plate	Trachea below stoma
10	14/M	Prolonged intubation for meningoencephalitis	Jackson’s tube (copper, zinc, nickel)	96	Junction between inner tube and neck plate	Carina
11	12/F	Bilateral abductor palsy	Fuller’s tube (copper and Zinc)	48	Just distal to the junction of two flanges	RMB

**Table:** 

**S.No**	**Factors causing fracture of the tracheostomy tube**	**Number (%)**
1.	Repeated boiling as the sterilization method	11 (100%)
2.	Irregular follow up	11 (100%)
3.	History of chronic cough	11 (100%)
4.	Lack of awareness about tracheostomy care	11 (100%)
5.	Prolonged usage	10 (90.9%)
6.	Stomal infection	10 (90.9%)
7.	Narrow stoma	9 (81.8%)
8.	Stomal granulation tissue	8 (72.7%)
9.	Type of tube a) Jackson’s tube b) Romson’s tube c) Fuller’s tube	5 (45.5%) 4 (36.4%) 2 (18.2%)
10.	Single tube usage Two tubes usage	7 (63.6%) 4 (36.4%)

None of the patients had suction machine at home. All patients had infrequent and irregular follow up and none of them were aware of the possibility of fracture and migration of TT if used for a long time. 

## Discussion

Fractured metallic or plastic TT presenting as a FB in the tracheobronchial tree is a rare complication of tracheostomy (9). Metallic TT are provided with an inner cannula which can be reinserted after cleaning out the blocked secretions and sterilization. Metallic tubes are rigid and less likely to fracture; hence they are preferred in older children, and in those who require prolonged use despite discomfort during neck movements. In younger children however, plastic tubes are preferred over metallic tubes because usage of an inner metal tube increases the airway resistance by reducing the available airway space within the tube. Plastic tubes are larger, pliable, snugly fitting to the shape of the trachea. The inert nature of the plastic tube and their smooth surface reduces the mucus adherence thereby favouring the suctioning process. The disadvantage of plastic tube is that they are costlier, and their maintenance is difficult as regular suctioning is required to remove the retained secretions. The incidence of fracture is more with plastic tubes compared to metallic tubes (5,6). The possible causes for the fracture of TT could be due to prolonged wear and tear of the tubes, repeated boiling of the tubes for sterilization purpose causing tube corrosion, alkaline nature of the tracheobronchial secretions, tissue reaction to the leached out chemicals from plastic tubes, repeated infection of the stoma and growth of granulation tissue between flanges of Fuller’s tube causes constant pressure on the tube thereby resulting in weakness of the joint site, irregular follow up and manufacturing defects in the tubes (7-9). In a study done by Apichart et al. they had compared two tracheostomy tube (silver plated brass and stainless steel tracheostomy tube) by mimicking the mechanical (Hot water and forced insertion of inner tube) and chemical stress (saliva) the tube undergo while in body, by immersing both the TT in pool of saliva for a period of 6 months. Both the inner and outer tube of brass showed erosion but the stainless steel remained unchanged (10). They concluded that stainless tubes are durable and resistant.

The fracture of the TT in our patients were probably because of a combination of these factors. In developing countries, same tracheostomy tubes are used for up to 5–8 years due to poor socio-economic status of majority of patients (11).

Other factors which predispose fracture of TT include the proportion and composition of materials in the tracheostomy tube and the manufacturing processes, prolonged contact of the TT with tracheobronchial secretions, the methods of TT care, repeated sterilization of the tubes and ageing due to prolonged usage. 

The recommended composition of the metallic tracheostomy tube is 45% silver, 15% copper, 24% cadmium and 16% zinc. Studies have shown that zinc is responsible for the high degree of corrosion (10,11). Corrosion is also due to the alkaline pH of secretions and repeated boiling. It has also been shown that Indian made metallic tubes are made of an alloy with very thin layer of silver plating, which wears out in two to three-week time (12). Prolonged exposures to these alkaline secretions result in greenish deposits and corrosion of the metallic tube, described as ‘‘season cracking’’(13). Newer modified metallic tubes are prepared from stainless steel consisting of steel and chromium which do not stain, corrode or rust as easily as ordinary steel. Another problem with regards to metal tubes are lack of uniformity in the diameter of inner tubes. The length of the inner tube as compared to the outer tube is also inconsistent. Projected part of the inner tube varied from 2mm to 4.5 mm, although the recommended projection is 2 mm (12,13). These defects could add on to the possible cause of tube fracture. The main manufacturing defect with regards to the plastic tubes as reviewed in literature was the separation of the cannula from its collar (12,13). This could be due to the liquefaction of the glue used for fusing the cannula with the collar by alkaline pH and the heat and humidity of tropical climate. Hence, to avoid such a problem, it is recommended to use tubes with a single unit, or the collared end of the tube should have a rim which can prevent the tube slippage when the glue gets liquefied (12). The weak areas of the tracheostomy tube are the junction between the tube and the neck plate, the distal end of the tube and the site of fenestration (14,15). In this study, the most common fracture site involved was at the junction between the tube and the neck plate in both metallic and plastic TT. The limitations of our study are that the exact physical dimension of the tube could not be measured as tubes were broken and tube from the same company and the same batch were not available, chemical analysis of the tubes were not done to find out the exact composition of TT and the small sample size due to rarity of this complication. Hence, large prospective studies with objective methods and with control groups are warranted for a better understanding of the causes for FTT. Based on the existing literature and our experience, we recommend the following suggestions which include regular inspection of the tubes for possible damage and reduction in flexibility due to repeated sterilization (16,17). Scheduled replacement of TT in long term tracheostomised patients is recommended (5,6). Tube wear and damage can be reduced by providing patients with two or three sets of inner TT (9). The inner cannula may be cleaned daily and frequent cleaning may be required depending upon the amount and nature of the patient’s secretion. Daily cleaning and dressing of the tracheostomy stoma site and regularly scheduled follow up with periodic inspection of the TTs is recommended. The patients and caregivers must be trained adequately regarding TT care and encouraged to use portable suction machine at home while using plastic tubes. They should be educated regarding possible complications before their discharge from the hospital. A periodic review of the TT care technique may be helpful.

## Conclusion

Fracture and migration of tracheostomy tube into tracheobronchial tree is a rare but avoidable complication of tracheostomy, which can be reduced by good tracheostomy care. The most essential part of the prevention is the need to educate the parents and primary care physicians about proper tracheostomy home care.

## References

[1] Okafor BC (1983). Fracture of tracheostomy tubes Pathogenesis and prevention. J Laryngol Otol.

[2] Piromchai P, Lertchanaruengrit P, Vatanasapt P, Ratanaanekchai T, Thanaviratananich S (2010). Fractured metallic tracheostomy tube in a child: a case report and review of the literature. J Med Case Rep.

[3] Majid AA (1989). Fractured silver tracheostomy tube: a case report and literature review. Singapore Med J.

[4] Gana PN, Takwoingi YM (2000). Fractured tracheostomy tubes in the tracheobronchial tree of a child. Int J Pediatr Otorhinolaryngol.

[5] Parida PK, Kalaiarasi R, Gopalakrishnan S, Saxena SK (2014). Fractured and migrated tracheostomy tube in the tracheobronchial tree. Int J Pediatr Otorhinolaryngol.

[6] Krempl GA, Otto RA (1999). Fracture at fenestration of synthetic tracheostomy tube resulting in a tracheobronchial airway foreign body. South Med J.

[7] Kakar PK, Saharia PS (1972). An unusual foreign body in the tracheo-bronchial tree. J Laryngol Otol.

[8] Kemper BI, Rosica N, Myers EN, Sparkman T (1972). Inner migration of the inner cannula: an unusual foreign body. Eye Ear Nose Throat Mon.

[9] Gupta SL, Swaminathan S, Ramya R, Parida S (2016). Fractured tracheostomy tube presenting as a foreign body in a paediatric patient. BMJ Case Rep.

[10] So-Ngern A, Boonsarngsuk V (2016). Fractured metallic tracheostomy tube: A rare complication of tracheostomy. Respir Med Case Rep.

[11] Otto RA, Davis W (1985). Tracheostomy tube fracture: an unusual etiology of upper respiratory airway obstruction. Laryngoscope.

[12] Chaturvedi VN, Gupta NB (1995). Study of size and manufacturing defects of various tracheostomy tubes. Indian J Otolaryngol Head Neck Surg.

[13] Bhargava MS, Bhat MS (1993). Broken tracheostomy introducer-an unusual tracheobronchial foreign body. J Laryngol Otol.

[14] Alvi A, Zahtz GD (1994). Fracture of a synthetic fenestrated tracheostomy tube: case report and review of the literature. Am J Otolaryngol.

[15] Lynrah ZA, Goyal S, Goyal A (2012). Fractured tracheostomy tube as foreign body bronchus: Our experience with three cases. Int J Pediatr Otorhinolaryngol.

[16] Sherman JM, Davis S, Albamonte-Petrick S (2000). Care of the child with a chronic tracheostomy. Am J Respir Crit Care Med.

[17] Fiske E (2004). Effective strategies to prepare infants and families for home tracheotomy care. Adv Neonatal Care.

